# Periodontal Responses to Augmented Corticotomy with Collagen Membrane Application during Orthodontic Buccal Tipping in Dogs

**DOI:** 10.1155/2014/873918

**Published:** 2014-09-09

**Authors:** Dong-Yeol Lee, Hyo-Won Ahn, Yeek Herr, Young-Hyuk Kwon, Seong-Hun Kim, Eun-Cheol Kim

**Affiliations:** ^1^Department of Periodontology, School of Dentistry, Kyung Hee University, No. 1, Hoegi-dong, Dongdaemun-gu, Seoul 130-701, Republic of Korea; ^2^Department of Oral and Maxillofacial Pathology and Research Center for Tooth and Periodontal Regeneration (MRC), School of Dentistry, Kyung Hee University, Seoul 130-701, Republic of Korea; ^3^Department of Orthodontics, School of Dentistry, Kyung Hee University, No. 1, Hoegi-dong, Dongdaemun-gu, Seoul 130-701, Republic of Korea

## Abstract

This prospective randomized split-mouth study was performed to examine the effects of absorbable collagen membrane (ACM) application in augmented corticotomy using deproteinized bovine bone mineral (DBBM), during orthodontic buccal tipping movement in the dog. After buccal circumscribing corticotomy and DBBM grafting into the decorticated area, flaps were repositioned and sutured on control sides. ACM was overlaid and secured with membrane tacks, on test sides only, and the flaps were repositioned and sutured. Closed coil springs were used to apply 200 g orthodontic force in the buccolingual direction on the second and third premolars, immediately after primary flap closure. The buccal tipping angles were 31.19 ± 14.60° and 28.12 ± 11.48° on the control and test sides, respectively. A mean of 79.5 ± 16.0% of the buccal bone wall was replaced by new bone on the control side, and on the test side 78.9 ± 19.5% was replaced. ACM application promoted an even bone surface. In conclusion, ACM application in augmented corticotomy using DBBM might stimulate periodontal tissue reestablishment, which is useful for rapid orthodontic treatment or guided bone regeneration. In particular, ACM could control the formation of mesenchymal matrix, facilitating an even bone surface.

## 1. Introduction

Augmented corticotomy, combining corticotomy and alveolar bone augmentation, is associated with favorable clinical outcomes in orthodontics. In particular, in Class III malocclusion, it promotes retention of the periodontal ligament and prevents bony dehiscence during mandibular anterior decompensation [1, 2]. The bone graft between the periosteum and the cortical surface functions as a scaffold for bone formation. In particular, alveolar bone thickness in the apical areas of the mandibular incisors increases significantly after bone grafting [2]. However, reports including histological observations of the periodontal reactions to augmented corticotomy using absorbable collagen membrane (ACM) are rare.

ACM is commonly used in dentistry, due to its biocompatibility and ability to promote wound healing [3]. Absorbable collagen barrier membranes inhibit migration of epithelial cells, promote attachment of new connective tissue, are not strongly antigenic, prevent blood loss by promoting platelet aggregation leading to early clot formation and wound stabilization, and do not require surgical removal [4, 5]. Collagen membranes may also facilitate primary wound closure via fibroblast chemotactic properties [5], even after membrane exposure [6]. Compared to nonabsorbable e-PTFE membranes, resorbable barriers allow for fewer exposures and therefore reduce the effects of infection on newly formed bone [4]. Use of collagen membranes in particular, with bone mineral as a support and space maintainer, has achieved predictable treatment outcomes [7–10].

ACM has properties comparable to those of nonabsorbable membrane, when used in guided tissue regeneration (GTR) [11, 12] and guided bone regeneration (GBR) [13, 14]. The use of a bone graft material in combination with collagen membrane improves clinical outcomes of intrabony defects [15, 16]. Therefore, application of a barrier membrane in augmented corticotomy could stabilize the graft material during healing and prevent bony dehiscence that can cause gingival recessions in soft tissue, ultimately improving bone regeneration potential.

In this study, we examined the effects of ACM application in augmented corticotomy with deproteinized bovine bone mineral (DBBM), during orthodontic buccal tipping movement in the dog.

## 2. Materials and Methods

### 2.1. Animals

The experimental protocols used in this study were approved by the Kyung Hee Medical Center Institutional Animal Care and Use Committee (KHMC-IACUC 11-021). We used a prospective, randomized, split-mouth study design in 2 male beagles, aged over 1 year and weighing 10–13 kg. The animals were caged individually with regulated light and temperature. They were fed a normal soft diet and had access to water* ad libitum*.

For the clinical and surgical procedures, the dogs were anesthetized with a mixture of tiletamine-zolazepam (5–10 mg/kg intramuscularly; Zoletil 100, Virbac, Carros, France) and xylazine (5 mg/kg intravenously using a catheter in an ear vessel; Rompun, Bayer Korea, Seoul, Republic of Korea). They were sacrificed under general anesthesia with an overdose of thiopental, 12 weeks after the surgery.

### 2.2. Clinical Examination

**Figure 1:**
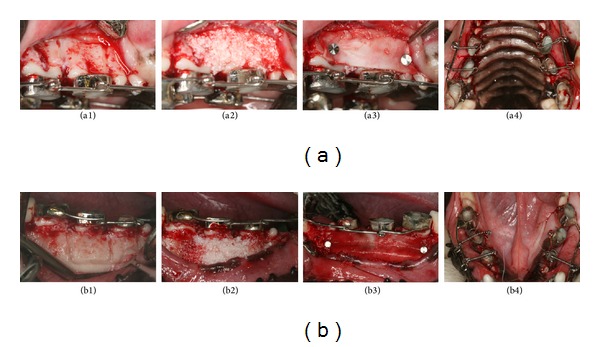
Surgical procedure in the (a) maxilla and (b) mandible. (1) Full-thickness flap reflection and buccal circumscribing corticotomy. (2) DBBM grafting on both the control and the test side. (3) ACM application with membrane tacks, on the test side only. (4) Primary flap closure and activation of the closed coil spring (200 g orthodontic force) for buccal tipping movement of the second and third premolars.

The experimental teeth were the second and third premolars of both maxillae and mandibles. Probing depth (PD) and the width of keratinized tissue (WKT) on the buccal sides of the second and third premolars were measured with a periodontal probe (Hu-Friedy, Chicago, IL, USA) at 3 sites (mesial, middle, and distal) on the buccal aspect before the surgery (baseline). Alginate impressions were made to fabricate study models. The canine and fourth premolar were banded for use as anchors, and thick wire (ø 0.9 mm) was welded to the buccal surface of the bands, which was subsequently used to locate the canine and fourth premolar. Lingual buttons were welded to the lingual surface of the band located on the second and third premolars (Figure 1).

### 2.3. Surgical Procedure

After the animals were anesthetized to fix the orthodontic appliances to the teeth, local anesthesia with 2% lidocaine solution (1 : 100,000 epinephrine; Lidocaine HCL, Huons, Seoul, Republic of Korea) was induced at the surgical sites. Control and test sides were randomly assigned in the mandible and maxilla. A total of 16 teeth were included in the study, 8 teeth each in the control and test sides. Intrasulcular incisions were made from the canines to the first molars, and full-thickness flaps were reflected. Circumscribing corticotomy was performed with a round bur (ø 1.5 mm) under sterile saline irrigation, and DBBM (Bio-Oss, Geistlich Biomaterials, Wolhusen, Switzerland) was grafted into the decorticated area. On control sides, flaps were repositioned and sutured. On test sides, ACM (Bio-Gide, Geistlich Biomaterials) was overlaid on the grafts and secured with membrane tacks (Membrane Pin, Dentium Co., Seoul, Republic of Korea), and then flaps were sutured. After primary flap closure, closed coil springs were activated to apply 200 g orthodontic force in the buccolingual direction and initiate immediate buccal tipping movement of the second and third premolars (Figure 1), in the control and test sides. The antibiotic gentamycin and the anti-inflammatory analgesic ketoprofen were intramuscularly administered twice daily for 6 days. Mechanical plaque control was performed once a week. PD and WKT were measured again when the animals were sacrificed.

### 2.4. Histological Examination

**Figure 2:**
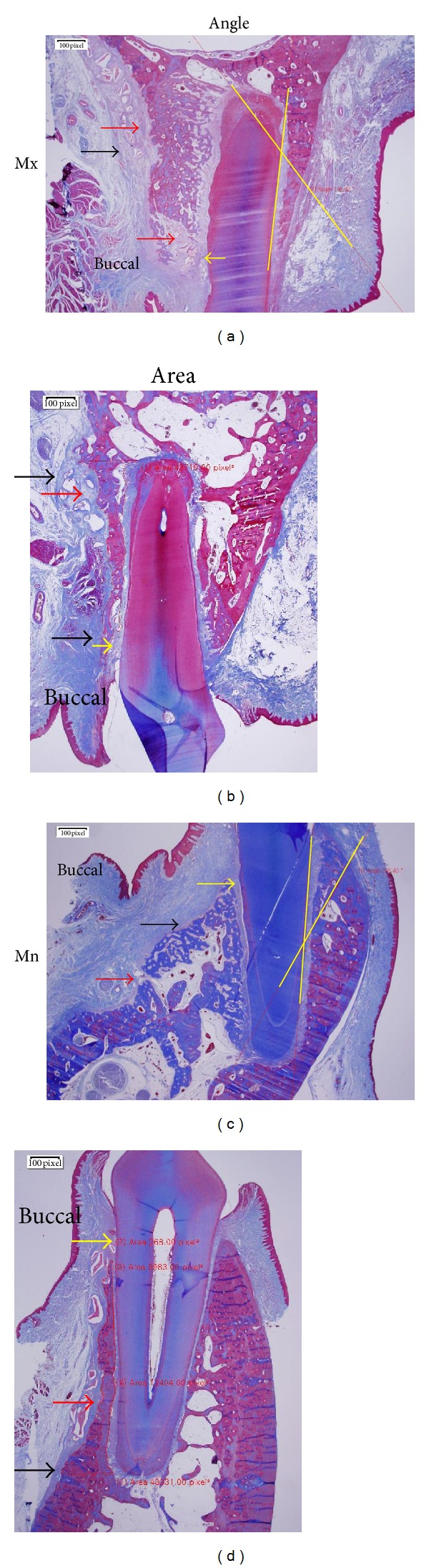
Representative micrographs for measuring the buccal tipping angle ((a), (c)) and bone area ((b), (d)) in the maxilla ((a), (b)) and mandible ((c), (d)). Red, yellow, and black arrows indicate the bone-derived, PDL-derived, and buccal mesenchymal matrices, respectively. Intersection of the yellow lines represents buccal tipping angle ((a), (c)). New bone area (%) was calculated by subtracting old bone area from total bone area. Masson's trichrome stain was used, and the original magnification was ×12.5.

The experimental sites were dissected, and retrieved block specimens were immersed in 10% neutral-buffered formalin for 14 days. Decalcification was performed by using 5% nitric acid for 6 days. Due to the large size of the block retrieved from the canine to the fourth premolar, 5% nitric acid was utilized for rapid decalcification, as it is a strong acid [17]. Notably, in a similar study designed for immunohistology, the use of EDTA or 10% aqueous or formic acid would be suitable [18]. However, as the aim of this study was not immunohistological analysis, due to time considerations, nitric acid was used to decalcify the retrieved block mass [19]. The specimens were then dehydrated through an ethanol series and embedded in paraffin. One slide was processed per experimental tooth. Buccolingual sections (5 *μ*m) were stained with Masson's trichrome. Histological examinations were performed under a light microscope (Olympus BX 51, Olympus, Tokyo, Japan) equipped with a DP21 camera. Each slide was photographed, and the resulting images were saved. Three examiners used imaging software (cellSens version 1.6, Olympus) to measure the buccal tipping angle (°) and bone area (%). The buccal tipping angle was measured from the reversal line of the lingual/palatal bone wall to the lingual/palatal root surface (Figures 2(a) and 2(c), yellow lines). New bone area was calculated by subtracting old bone area from total bone area (%) on the buccal side from crest to apex level. Grafted particles embedded in and bridged with new bone were included in the calculation, but floating particles in connective tissue were excluded (Figure 2).

### 2.5. Statistical Analysis

One slide was obtained per tooth. As the experimental areas were P2 and P3, a total of 16 slides were obtained. The control and test groups each included 8 slides. All data were analyzed using commercially available software (SPSS version 18.0, SPSS, Inc., Chicago, IL, USA). The Wilcoxon test was used to compare the baseline and postsurgical values. The Mann-Whitney *U* test was used to compare the test and control sides. The *α* error was set at 0.05. Interexaminer differences were evaluated with the intraclass correlation coefficient (ICC).

## 3. Results

### 3.1. Clinical and Histomorphometric Findings

All the experimental sites showed uneventful healing and minimal, if any, inflammatory signs. PD increased after the surgery on both the test (by 0.704 mm; *P* = 0.001) and the control (by 0.136 mm; *P* = 0.011) sides. The increase in PD was more prominent on test sides than control sides. On the control sides, the WKT reduced significantly by 0.455 mm (*P* = 0.028). However, on test sides, the WKT reduction of 0.250 mm was not significant (*P* = 0.410) (Table 1). The ICCs for the buccal tipping angle and bone area measurements were 0.997 and 0.956, respectively (*P* < 0.001 in both cases). The buccal tipping angles were 31.19° ± 14.60° and 28.12° ±11.48° on the control and test sides, respectively, and this difference was not statistically significant (*P* = 0.406). The new bone area was 79.5 ± 16.0% on the control side, and on the test side it was 78.9 ± 19.5%, not a statistically significant difference (*P* = 0.949) (Table 1). Approximately 79% of buccal bone wall was reformed. There were no statistically significant differences in new bone formation at the buccal wall, or in tipping movement, between the groups.

### 3.2. Histological Observations

Connective tissue within the buccal bone was designated bone-derived mesenchymal matrix (Figure 2, red arrow) and that in the periodontal ligament (PDL) was designated PDL-derived mesenchymal matrix (Figure 2, yellow arrow). Thick, dense connective tissue covering the buccal bone surface was designated buccal mesenchymal matrix (Figure 2, black arrow).

**Figure 3:**
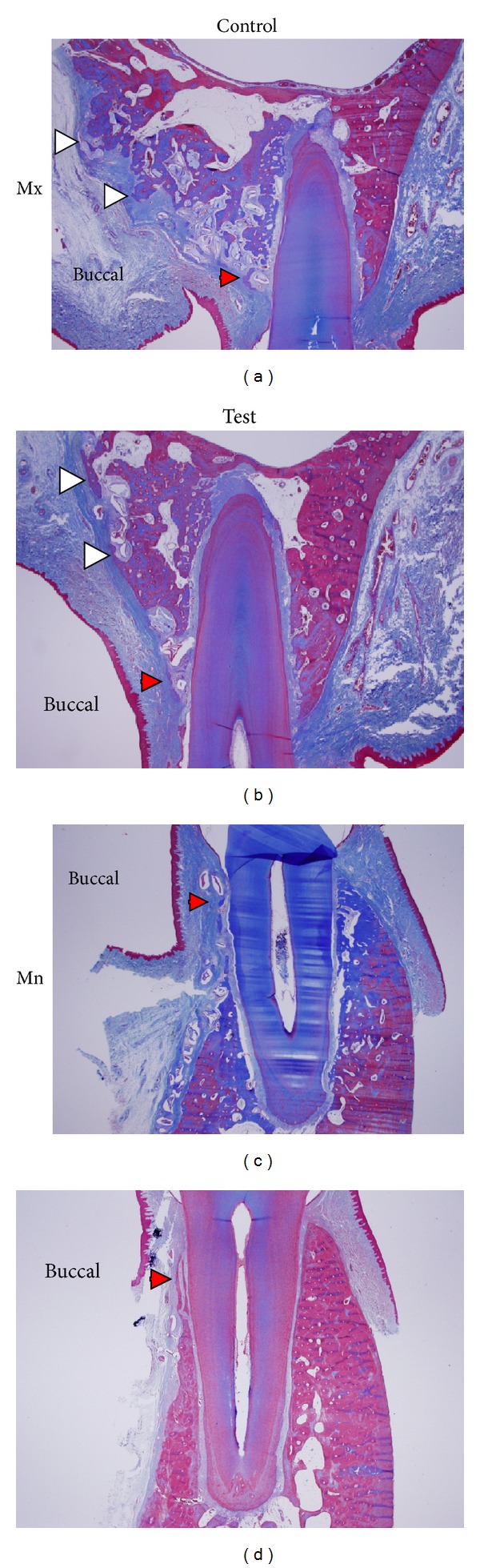
Micrographs of buccolingual sections from the control ((a), (c)) and test sides ((b), (d)) in the maxilla ((a), (b)) and mandible ((c), (d)). Red arrowheads indicate bone formation over DBBM particles in the crest area. White arrowheads in (a) (control) indicate exophytic new bone formation from the buccal bone wall by bone-derived mesenchymal matrix that formed irregular bone surfaces. White arrowheads in (b) (test) indicate new bone formation from the buccal bone wall by bone-derived mesenchymal matrix that formed even bone surfaces. Masson's trichrome stain was used, and the original magnification was ×12.5.

**Figure 4:**
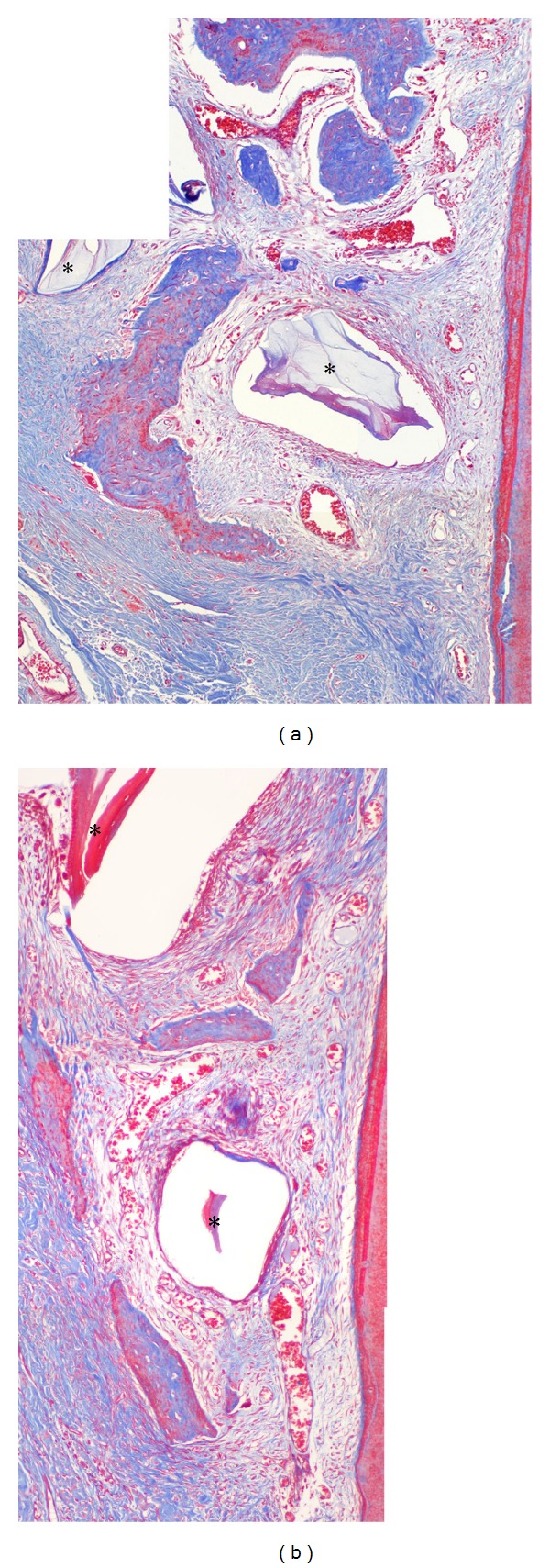
New bone formation in the crest area. (a) Magnification of red arrow head in Figure 3(a) (control). (b) Magnification of red arrowhead in Figure 3(b) (test). Newly formed bone islands covered the grafted DBBM particle (∗) along the root surfaces in PDL-derived mesenchymal matrix. R: root. Masson's trichrome stain was used, and the original magnification was ×100.

**Figure 5:**
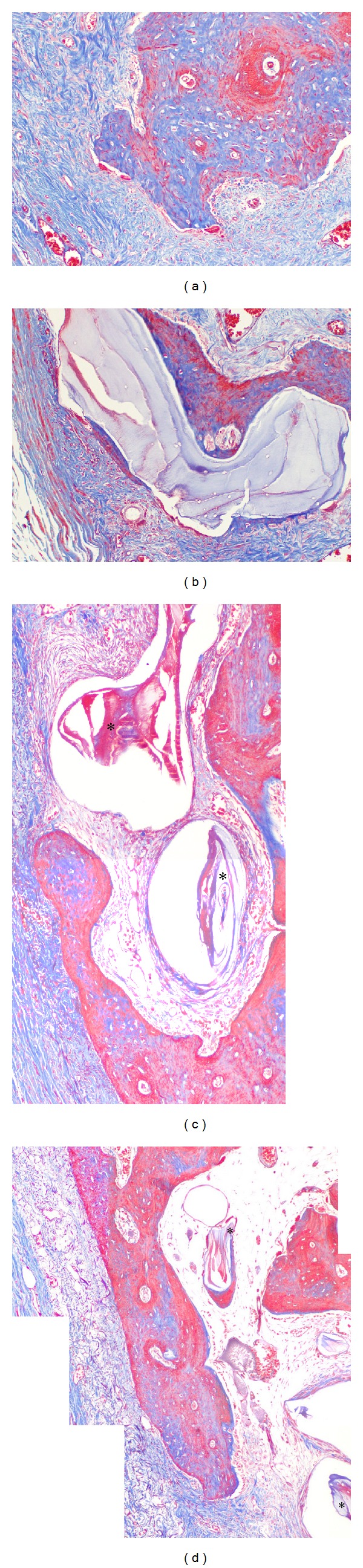
New bone formation in the apical area. (a), (b) Magnification of white arrowheads in Figure 3(a) (control). Exophytic bone formation from the bone surface by bone-derived mesenchymal matrix formed irregular bone surfaces. (c), (d) Magnification of white arrowheads in Figure 3(b) (test). ACM restricted the flow or extension of bone-derived mesenchymal matrix and resulted in an even surface. The grafted DBBM particles (∗) which were encapsulated by matrix or connected to newly formed bone are indicated. Masson's trichrome stain was used, and the original magnification was ×100.

Bone formation was substantial in the bone-derived mesenchymal matrix (Figure 3). Some areas did not show DBBM particles. However, most of the particles were embedded in or bridged with new bone and/or encapsulated by the bone-derived and/or buccal mesenchymal matrix. Two features were prominent. One was the considerable amount of bone formation from the bone-derived mesenchymal matrix (Figure 3, white arrowheads), and the other was reformation of the buccal bone crest from bone-derived and/or PDL-derived mesenchymal matrix in the coronal direction along the root surfaces (Figure 3, red arrowheads, Figures 4(a) and 4(b)). In this study, original buccal bone at the crest was not seen, and encapsulated graft particles were embedded in bone-/PDL-derived mesenchymal matrices (Figures 4(a) and 4(b)). Bone modeling was not localized; it was apparent throughout the buccolingual alveolar and basal bone. The buccal mesenchymal matrix covered the buccal bone surface and seemed to play a role in periosteum. Enlarged bone marrow filled with fat tissue was also a distinguishing phenomenon. The maxillary buccal bone surface in the middle/apical area was flatter on the test side than on the control side (Figure 3(b), white arrowheads, Figure 5). Gingival recession was absent, but root surface resorption was sometimes observed.

## 4. Discussion

It has been assumed that, in alveolar remodeling during orthodontic tooth movement, the amounts of bone resorption and formation are equal. However, recent computed tomography (CT) studies have shown that alveolar bone thickness decreases in the direction of tooth movement [20–22]. The remodeling capacity of alveolar bone cannot compensate for bone loss in every case. Once the cortical plate is fenestrated, the buccal root surface becomes devoid of cortical bone [20–22], and subsequent osteogenesis is insufficient to cover the root surface completely. CT scans have not shown newly formed cortical plate in patients who develop fenestration [23, 24]. In addition, histological studies have not demonstrated regeneration of the cortical plate [25, 26]. Therefore, orthodontic tooth movement beyond the alveolar housing can cause periodontal problems such as fenestration, dehiscence of the buccal cortical plate in hard tissue, and gingival recession in soft tissue. Our results, however, show that augmented corticotomy using DBBM combined with ACM application enables reformation of buccal bone crest on the pressure side, such that buccal soft tissue height could be maintained despite excessive buccal tipping movement. In our study, PD was increased slightly but statistically significantly. WKT was slightly reduced; however, we consider the amount of that reduction negligible from a clinical point of view. Clinical attachment level (CAL) was not measured, but the extent of the change in CAL was thought to be insignificant clinically.

The ability of bone to adapt to mechanical loads is brought about by continuous bone resorption and formation. If these processes occur at different locations, bone morphology can be altered. Frost [27] termed this phenomenon “bone modeling.” If bone resorption and formation are balanced, old bone is continuously replaced by new bone, the mechanical integrity of the bone is maintained, and no morphological changes occur. Frost [28, 29] termed this lack of morphological changes “bone remodeling.” As shown in Figures 3 and 4, bone formation from the PDL-/bone-derived mesenchymal matrix recovered the buccal bone crest, and newly formed bone islands covered graft particles (Figure 4) and old bone (Figure 3(d), red arrowhead). The original buccal bone plate and most of the DBBM graft particles were resorbed. The expansion of mesenchymal matrices enabled reformation of bone crest and formed new bone on the buccal side. These phenomena are thought to represent “bone modeling,” which maintains soft tissue height at the buccal bone crest. In this bone modeling, periodontal tissue reestablishment was demonstrated. The effect of membrane application was prominent in the middle/apical area of buccal bone (Figures 3 and 5). This suggests that ACM can control the shape of the bone surface, as well as performing its primary function.

Yaffe et al. [30] investigated regional acceleratory phenomenon (RAP) by using radiological methods. They reported enlarged bone marrow and striking resorption of the cortical bone, both on the surface and in the alveolar bone proper, on the buccal aspect. Our study yielded the same histological finding. In addition, the expansion of bone marrow and buccal bone by the bone-derived mesenchymal matrix is thought to contribute to bone modeling and remodeling.

Some bone formation or modeling at the buccal crest might function as a compensatory mechanism [31]. Orthodontic tooth movement is a stimulating factor for bone apposition [32, 33]. However, whether the stimulating factor for compensatory bone formation was corticotomy or orthodontic force was not clear in this study. Further research is required to elucidate the stimulating factors in this context.

This study demonstrated an optimal response to applied forces, because the response was mediated by the PDL, spongiosa [34], and periosteum. More active and extensive bone modeling and remodeling suggest that accelerated tooth movement associated with augmented corticotomy is due to increased bone turnover and RAP [35], as shown by our results. Further, Nowzari et al. [36] reported that the alveolar ridge maintains its original thickness and configuration despite buccal tipping movement. Our study also demonstrated that the alveolar ridge width does not decrease, and the buccal bone crest is maintained despite excessive buccal tipping movement. Moreover, Machado et al. [37] reported a reduction of 1.1 mm in apical root resorption of the maxillary central incisors, in comparison with traditional orthodontics. However, in the current study, only root surface resorption was found at some buccal and apical pressure areas.

Wilcko et al. [38] suggested that when the relatively thin alveolar housing over the root surface undergoes demineralization, the remaining collagenous soft tissue matrix of the bone could be readily transported with the root surface, in the direction of movement, a phenomenon termed “bone matrix transportation.” When retained in the desired position, the matrix is remineralized. Our study demonstrated new bone in almost 79% of the buccal bone wall at 12 weeks after augmented corticotomy with DBBM, irrespective of the application of ACM. Nevertheless, whether bone matrix transportation occurred is not clear, and its mechanism should be investigated. This phenomenon could be part of bone modeling/remodeling and activation. In the grafted areas, new bone emerged from the existing bone surface and bridged the DBBM particles in the bone- or PDL-derived mesenchymal matrix. Bone modeling and remodeling might have occurred simultaneously and resulted in graft entrapment and resorption over the 12 weeks. The phenomena associated with augmented corticotomy with DBBM may be multifactorial responses and may not be caused by bone matrix transportation.

## 5. Conclusions

Buccal tipping angle and new bone formation in the buccal wall are not directly affected by the use of ACM in augmented corticotomy. However, the use of ACM in augmented corticotomy can promote an even buccal bone surface morphology. Augmented corticotomy using DBBM combined with ACM application may stimulate periodontal tissue reestablishment, and intact PDL and periosteum are prerequisites for optimal therapeutic outcomes. It may be particularly suitable for rapid orthodontic treatment or GBR.

## Figures and Tables

**Table 1:** Probing depth, width of keratinized tissue, buccal tipping angle, and new bone area.

		PD (mm)	WKT (mm)	Angle (°)	NB (%)
Control	Baseline	1.636 ± 0.492	3.955 ± 0.461^#^	31.19 ± 14.60	79.5 ± 16.0
	12 weeks	1.773 ± 0.685^†^	3.500 ± 0.802^#^		

Test	Baseline	1.682 ± 0.451*	4.000 ± 0.787	28.12 ± 11.48	78.9 ± 19.5
	12 weeks	2.386 ± 0.755^∗,†^	3.750 ± 0.935		

^∗†#^ *P* < 0.05.
